# Low‐dose oestrogen–progestin associated pulmonary infarction mimicking pneumonia and pleurisy

**DOI:** 10.1002/rcr2.833

**Published:** 2021-08-17

**Authors:** Kyoko Gocho, Shinnosuke Kitazawa, Shinya Matsushita, Nobuyuki Hamanaka

**Affiliations:** ^1^ Department of Respiratory Medicine Saiseikai Yokohamashi Tobu Hospital Yokohama Japan

**Keywords:** central lucency, low‐dose oestrogen–progestin, pulmonary infarction, pulmonary thromboembolism, venous thromboembolism

## Abstract

A 28‐year‐old woman with a history of treatment with a low‐dose oestrogen–progestin (LEP) formulation presented to our hospital due to right chest pain. She had just been discharged from another hospital for pneumonia and pleurisy which had improved with antibiotics. Contrast‐enhanced computed tomography (CT) revealed bilateral pulmonary emboli corresponding to the peripheral consolidations. The pulmonary emboli indicated that the peripheral consolidation was due to pulmonary infarction (PI). No aetiological factors were identified except for the history of LEP therapy. Although the typical CT images of PI are consolidations in the peripheral area, these finding are non‐specific for PI. This case of PI was misdiagnosed as infection because of response to antibiotics and similar CT findings. Therefore, careful evaluation of the patient history and clinical findings are imperative for accurate diagnosis. Venous thromboembolism can occur frequently around 3 months after the start of LEP treatment.

## Graphical abstract

We report a case of pulmonary infarction in a young woman with a history of treatment with a low‐dose oestrogen–progestin (LEP) formulation that was misdiagnosed as infection because of response to antibiotics and similar computed tomography findings. Therefore, careful evaluation of the patient history and clinical findings are imperative for accurate diagnosis. Venous thromboembolism can occur frequently around 3 months after the start of LEP treatment.

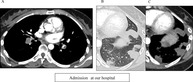

## INTRODUCTION

Pulmonary infarction (PI) is a relatively rare condition that may occur as a complication of pulmonary thromboembolism (PTE). Approximately 16% of patients with PTE may have associated PI.^1^ Owing to the lack of specific computed tomography (CT) findings of PI, it is important to distinguish PI from other diseases including infectious diseases.

## CASE REPORT

A 28‐year‐old Japanese woman presented to our hospital due to right chest pain. She had just been discharged from another hospital the day before presenting at our hospital. There was no history of smoking, respiratory disease or cardiovascular disease. She had received low‐dose oestrogen–progestin (LEP) therapy for dysmenorrhoea for 3 months and had stopped taking it 1 month ago.

On examination, her temperature was 37.6°C, pulse rate was 70 beats per minute, blood pressure was 140/74 mmHg and respiratory rate was 25 per minute. On chest auscultation, there were no crackles in the lung fields or heart murmur. There was no swelling or pain in the lower limbs.

Laboratory investigations revealed almost normal results with no inflammation except for elevated d‐dimer (6.9 μg/ml) and hypoxaemia on arterial blood gas analysis (partial pressure of oxygen [PaO_2]_ 56 mmHg). Brain natriuretic peptide and troponin T levels were not elevated.

**FIGURE 1:**
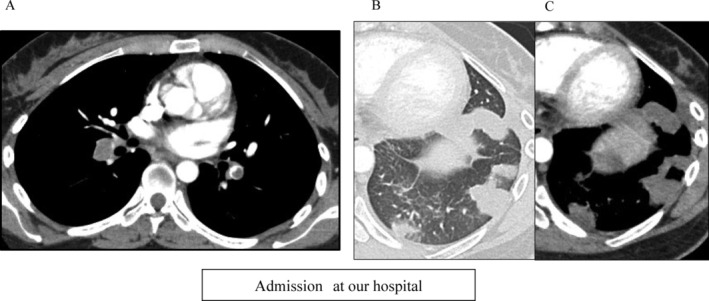
Contrast‐enhanced computed tomography images obtained at our hospital. (A) Mediastinal window setting. Pulmonary emboli are seen in the bilateral inferior pulmonary artery. (B) Lung window setting. Some well‐demarcated air space consolidation lesions are seen in the left lower lobe. One of the consolidation lesions shows central lucency. (C) Mediastinal window setting. The central lucency is better seen on mediastinal window setting

Contrast‐enhanced CT of the chest showed multiple pulmonary emboli in the pulmonary arteries (Figure 1A). There were some well‐demarcated subpleural air space consolidations without CT enhancement in the left lower lobe along with mild pleural effusion (Figure 1B,C). Pelvic CT revealed a thrombus in the left common iliac vein.

Electrocardiogram showed no T wave inversion in the anterior precordial lead or right axis deviation. There was no right ventricle enlargement suggestive of pulmonary hypertension or right heart failure in the cardiac ultrasonography.

**FIGURE 2:**
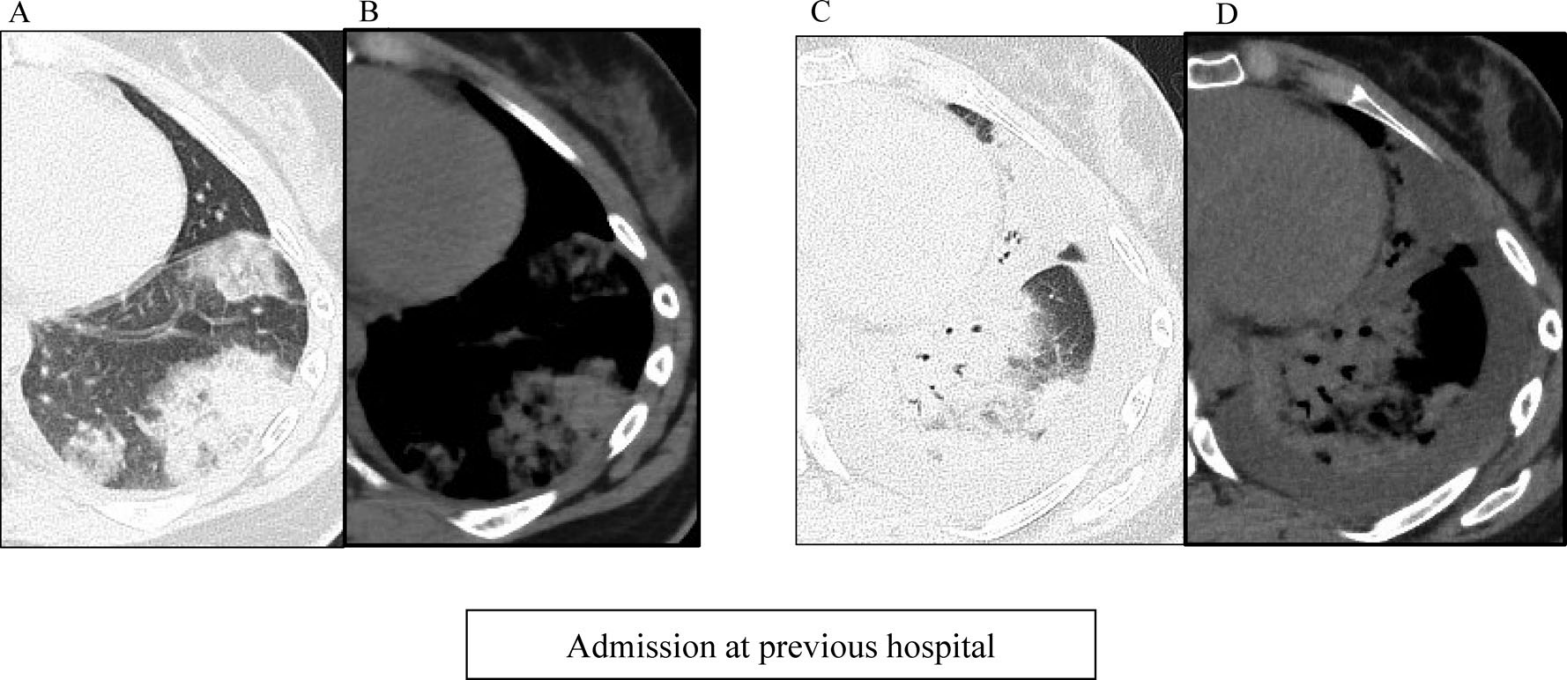
Plain computed tomography images obtained at the previous hospital. (A and B) Images obtained on the first day of admission at the previous hospital. (A) Lung window setting. Poorly demarcated patchy consolidation is seen in the left lower lobe. (B) Mediastinal window setting. Mild pleural effusion is seen. (C and D) Images obtained on the fourth day of admission at the previous hospital. (C) Lung window setting. (D) Mediastinal window setting. Pleural effusion has increased leading to atelectasis of the left lower lobes

The consolidation lesions in CT were consistent with the lung shadows which had led to a diagnosis of infectious pneumonia in the previous hospital. Two weeks previously, she had visited the previous hospital due to left back pain. She had elevated C‐reactive protein (12 mg/dl), white blood cells (1100/μl) and d‐dimer (9.4 μg/ml). Initial CT was non‐contrast CT and showed poorly demarcated subpleural consolidations in the left lower lobe (Figure 2A,B). She was diagnosed with pneumonia and pleurisy at the previous hospital. Although there was transient atelectasis in the left lower lobe due to increased pleural effusion, antibiotic treatment led to alleviation of inflammation, pleural effusion and back pain except for the high d‐dimer levels (Figure 2C,D).

In our hospital, the consolidations were diagnosed as PI based on elevated d‐dimer, symptoms of pleurisy and pleural effusion, and CT images obtained from the previous hospital. All consolidation lesions were located in the territories of the embolized arteries. She was treated with rivaroxaban. Detailed evaluation revealed no other apparent cause for the PTE and PI except for her past history of LEP therapy. The patient had no past and family history of thrombosis. There was no deficiency of antithrombin III, protein C or protein S. The lupus anticoagulant and IgG‐type anticardiolipin antibody were negative. Moreover, there were no signs of collagen disorders. On the 18th day of hospitalization, she was discharged from our hospital and is currently receiving outpatient treatment.

## DISCUSSION

The rarity of PI may be attributable to the characteristics of the pulmonary vascular system. Pulmonary vascular system has a dual blood supplies, the pulmonary artery and the bronchial artery. However, there is anastomosis of the two vessels in the lung periphery around the subpleural region. PI develops when embolism and obstruction occur in the peripheral pulmonary artery beyond the area of dual blood supply within the bronchial artery.

LEP is one of the typical causes of abnormal hypercoagulability. The typical risks of venous thromboembolism (VTE) are old age and obesity, and VTE is most likely to occur during the first 3 months of LEP use.^2^ Our patient was a young non‐obese woman; however, she developed VTE just after 3 months of treatment.

In previous studies, PI associated with PTE was found more likely to occur in the elderly and those with heart disease.^3^ However, recent research has revealed an opposite trend. Miniati et al. reported that patients with PI were significantly younger than those without PI, and had a much lower prevalence of heart diseases.^4^ Islam et al. hypothesized that high incidence for younger patients is attributable to the protective effect of the collateral bronchopulmonary vessels that develop in the elderly due to concomitant chronic cardiopulmonary disease.^1^ PI often presents with symptoms of pleuritis such as chest pain, fever and pleural effusion, which makes it difficult to distinguish from infection. Several reports have described cases of PI that required differentiation from infection. In our case, the main pathological conditions were PTE and PI at initial visit. We speculate that the infection sign was due to a secondary infection associated with ischaemia, bleeding and oedema of the infarct lesion. Because the infarction resolved spontaneously from the acute phase and antibiotics were effective for the secondary infection, we think that the clinical findings improved gradually. Her back pain might be attributed to pleural pain due to PI.

On chest CT images, the typical findings of PI are consolidation in the peripheral area with central lucency, thickening of vessels leading to apex, absence of air bronchogram and pleural effusion.^5^ However, none of these findings are specific for PI. In addition, consolidation of PI can diminish in size over time accompanied by scarring; imaging signs of PI may persist for 10 weeks.^5^ In our case, at the previous hospitalization, the PI appeared as a poorly demarcated bubble‐like consolidation without air bronchogram, which changed to atelectasis with pleural effusion 5 days later. Two weeks after the first admission, the pleural effusion had resolved and the consolidation had diminished in size and changed to well‐demarcated consolidation with central lucency. Eventually, at 3 months after the first visit, there was a slight residual scar.

It is necessary to recognize that VTE can occur frequently around 3 months after the start of LEP treatment. PI does not have any specific clinical and imaging findings; however, it is a potentially fatal disease. Therefore, careful evaluation of the patient history, clinical findings and a high suspicion index are a key imperative for accurate diagnosis.

## CONFLICT OF INTEREST

None declared.

## AUTHOR CONTRIBUTION

All authors revised the manuscript, approved the manuscript to be published and agreed to be part of all aspects of the work in ensuring that questions related to the accuracy or integrity of any part of the work are appropriately investigated and resolved.

## ETHICS STATEMENT

Appropriate written informed consent was obtained for publication of this case report and accompanying images.
